# Place-Based Disparities Among Nursing Homes During the COVID-19 Pandemic: A Systematic Literature Review

**DOI:** 10.1089/heq.2024.0091

**Published:** 2025-01-22

**Authors:** Erica Gigas, Nicholas O’Neel, Lorinda A. Coombs, Jamie Conklin, Margaret Chamberlain Wilmoth, Baiming Zou, Patricia Schmidt, Saif Khairat

**Affiliations:** ^1^ UNC Chapel Hill, Chapel Hill, North Carolina, USA.; ^2^ FCNSI Tripler Army Medical Center, Honolulu, Hawaii, USA.; ^3^ Brooke Army Medical Center, Fort Sam Houston, Texas, USA.

**Keywords:** nursing homes, health disparities, COVID-19, place-based disparities

## Abstract

**Introduction:**

Disasters have disproportionately impacted nursing home (NH) residents. COVID-19 impacted NH more so than the community-dwelling population, but there was much variation in mortality rates among NH residents. These disparities have been studied, but place-based disparities have received less attention. Place-based disparities are differences in health due to physical location, including factors like rurality, local socioeconomic conditions, and the physical environment.

**Methods:**

We searched three databases for peer-reviewed studies of place-based factors associated with mortality in U.S. NHs during the COVID-19 pandemic, ending in January 2024. Data were organized using the National Institute on Minority Health and Health Disparities research framework.

**Results:**

We identified 27 articles that included individual, interpersonal, community, and societal place-based factors associated with mortality during the pandemic. Differences in mortality were related to local community socioeconomic factors, staff neighborhood socioeconomic factors, urbanity, community viral spread, and state-level factors, including political leaning and social distancing policies. Rurality was associated with lower mortality but was also associated with racial disparities.

**Discussion:**

Place-based disparities at the individual, organizational, community, and societal levels were identified. Rurality and local COVID-19 spread were the most commonly studied place-based factors associated with NH deaths during the pandemic. Neighborhood factors may be most impactful through the impact on NH staff. Racial disparities were linked with location, highlighting the effects of historical systemic racism on NHs. Policies to protect NH residents during disasters must be sensitive to local characteristics.

## Introduction

Disaster planning for nursing homes (NHs) has long been a challenge in the United States. For example, Hurricane Katrina had disproportionately negative impacts on NH residents.^1^ This problem resurfaced during the recent COVID-19 pandemic. In both examples, the NH location played a role in vulnerability. Modern disasters like Hurricane Katrina and the COVID-19 pandemic have had a disproportionate impact on NH residents, who are more vulnerable to both the events and the consequences.^2^ During the COVID-19 pandemic, NH residents were 23 times more likely to die of COVID-19 than community-dwelling older adults.^3^ NH deaths due to COVID-19 have received much scientific and media attention, but there has been less focus on disparities among NHs. Health disparities are measurable differences among population groups resulting from racial, social, economic, ethnic, geographic, or other factors.^4^ Understanding outcomes related to the physical location of the NH, for example, placed-based disparities, is essential for designing effective policies, resource allocation, and potential interventions.^5^

Studies of NH disparities have identified various factors related to health outcomes. Community spread of COVID-19 (a measure of COVID-19 cases in a geographic area) is the most impactful variable for predicting COVID-19 cases and mortality among NHs.^6^ Other community-related factors, such as, rurality, social vulnerability, or proximity to primary care, can impact health outcomes in different care settings. However, whether these factors are equally important for NH residents needs to be clarified.^7,8^ Place-based factors may impact NH mortalities related to structural, systemic inequities and other social determinants of health.^9,10^

A previous literature review of factors associated with NH disparities during the COVID-19 pandemic identified studies published during the first half of the pandemic (2020–July 2021). The prior review focused on NH characteristics rather than place-based characteristics;^4^ a systematic literature review focused on place-based disparities is needed because we are investigating geographic factors.

Our research question was, what place-based factors for NHs are associated with mortality disparities during the COVID-19 pandemic? The purpose of this study was to identify place-based factors that significantly impacted NH mortality to inform future policies and interventions for NHs during disasters.

## Methods

A systematic literature review was conducted to synthesize community and geographic factors associated with disparities in NH health outcomes. Several published papers have reported conflicting results on this topic. The review was conducted according to the PRISMA (Preferred Reporting Items for Systematic Reviews and Meta-Analyses) guidelines. The team worked from a protocol that was not registered.

### Data sources and search strategy

A search strategy was developed in collaboration with a health services librarian. Search terms were refined through iterative searches using keywords for mortality, deaths, NHs, skilled nursing facilities, and COVID-19. The databases included in the search were PubMed, Scopus, and CINAHL Plus with Full Text (EBSCO*host*) from papers published from 2020 through January 29, 2024. We searched for subject headings and keywords related to COVID-19, NHs, and mortality rates.

### Eligibility criteria and study selection

Studies were included in the review if they were published in English or had an English language abstract available since the focus was on NHs in the United States. Additionally, the reviewing authors were monolingual English speakers. Any quantitative study that used NH mortality as an outcome measure was included in the review. All long-term care, assisted living, and NH facilities were included.^6^ Mortality outcome measures included any measure of deaths, including death from COVID or other causes, excess deaths, counts of deaths, or likelihood of deaths. We focused on mortality measures only because deaths are a key indicator of the impact of the pandemic and the most severe indicator of health disparities. Papers needed to include place-based independent variables—any independent variables related to the NH’s geographic location.

Exclusion criteria were papers that did not include primary data analysis, studies conducted outside the United States (due to the unique health care system), and studies that focused only on outbreaks, rate of spread, or vaccination rates. Studies that used different outcome measures (such as state-level mortality rates) were also excluded.

Two authors screened the titles and abstracts of studies that met the inclusion criteria, and the same two authors (E.G. and N.O.N.) did the full-text screening. A third author (S.K.) resolved disagreements. The entire process was conducted using Covidence software.

### Data extraction process

One reviewer, in collaboration with two mentors, developed a structured data-charting tool that was piloted in three studies. A single reviewer extracted all raw data. During the abstraction process, if a new category or outcome was identified, the data abstraction tool was updated, and studies previously abstracted were re-reviewed. The data items extracted from the full-text articles included: (1) study design details, (2) sample characteristics, (3) data sources, (4) data analysis methods, (5) significant geographic/community-independent variables and their relationships, and (6) all reported outcomes.

### Data synthesis

The authors created a data matrix using relevant items from the data abstraction tool. Study characteristics were included and the synthesis focused on identifying place-based disparity variables in the studies and their relationships to COVID-19 mortality in NHs. Because we were interested in health disparities, we used the National Institute on Minority Health and Health Disparities to organize our results with the corresponding levels of interest in the framework.^11^ The framework categorizes levels of influence on health as individual, organizational/interpersonal, community, and societal. All mortality outcomes in our study were measured at the organizational level.

## Results

**FIG. 1.**
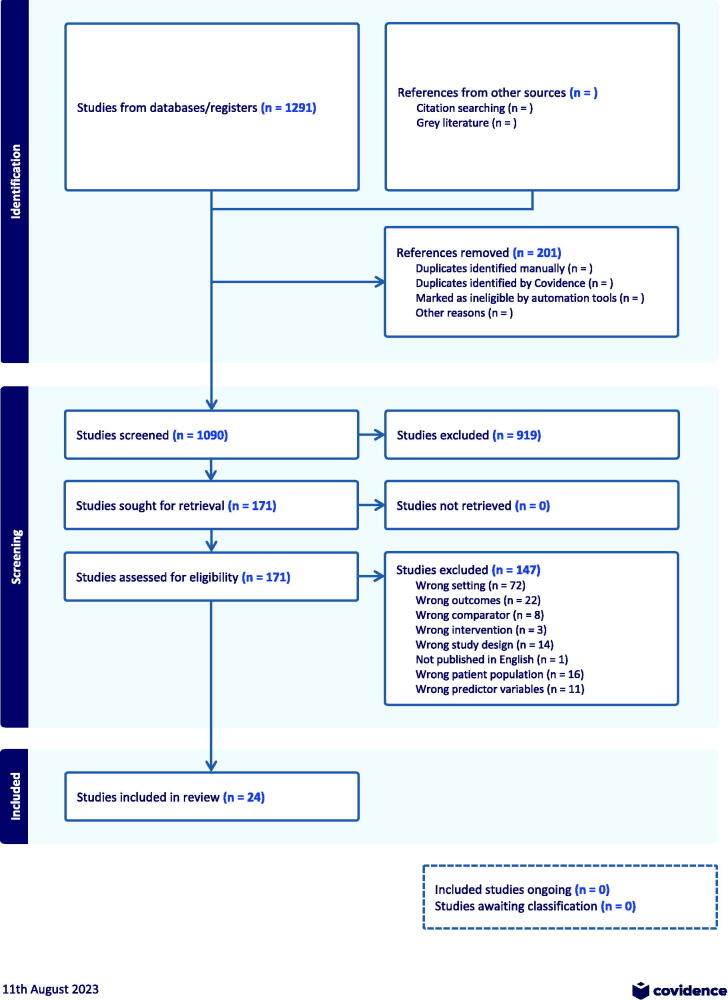
Nursing homes, COVID-19, and mortality.

The initial search yielded 1692 unique studies. The full-text review included 194 studies, of which 27 met the criteria (Fig. 1). Of the 27 studies, 6 used a longitudinal design. Seven studies used state-reported COVID-19 metrics, whereas all others used CMS COVID-19 data.

### Sample characteristics

Sample sizes ranged from 103 to 15,130 facilities. Eighteen studies included NHs in 50 states,^12–29^ four included individual states,^8,30–32^ four included multiple states,^21,33–35^ and one included a single county.^36^ One study included only assisted living facilities^33^; all others included NHs, assisted living facilities, and skilled nursing facilities (see Table 1).

**Table 1.** Study Characteristics

Study	Sample size	States	Time period	COVID-19 data sources	Study purpose	Outcomes
Temkin-Greener et al.^33^	3994	7	March 2020- May 29, 2020	State reports	Variations in COVID-19 deaths and relationship with “key assisted living characteristics”	Binary variable: was there a COVID-19 resident death in the facility?
Dean et al.^30^	355	NY	March 2020- May 31, 2020	State reports	Association between presence of a health care worker union and COVID-19 mortality rates in NH in NY	COVID-19 deaths/ total number of beds in the facility, reported as a percentage
LeRose et al.^8^	103	MI	March 2020-May 20, 2020	State report	Relationship between SVI and COVID-19 mortality rate	Number of COVID-19 deaths per bed
Li et al.^37^	12,576	50	25 May 2020–31 May 2020	NY Times reports, CMS report	Relationship between resident race/ethnicity and weekly resident COVID-19 deaths	Number of new COVID-19 deaths among residents, reported as 3 levels
Li et al.^31^	211	CT	April 13, 2020-June 19, 2020	State report	Relationship between resident race/ethnicity and COVID-19 deaths over time	Cumulative number (count) of laboratory-confirmed COVID-19 deaths
Gorges and Konetzka^38^	8626	50	June 25, 2020	CMS	Relationship between staffing levels and COVID-19 deaths	Count of deaths from COVID-19
Travers et al.^15^	11,587	50	Through 9 July 2020	CMS	Effect of structural bias and urbanicity/rurality on the relationship between resident race and COVID-19 deaths	Mortality rate, COVID-19 deaths/ bed days
Shen^34^	6,132	18	Through 10 July 2020	State reports	Relationship between characteristics of the residential neighborhoods of nursing home staff and COVID-19 mortality rates	Mortality rate: COVID-19 deaths/100 patients
Cai et al.^28^	13,123	50	7 June 2020, 23 August 2020	CMS	Association between racial/ethnic compositions of NH and their communities were associated with likelihood of COVID-19 deaths	Binary variable: was there a COVID-19 resident death in the facility?
Li et al.^17^	14,046	50	25 May 2020-August 23, 2020	CMS	Relationship between changes in state strength of social distancing restrictions and COVID and non-COVID resident deaths	More than one COVID-19 death per week; more than 1 non-COVID-19 death per week; counts of both COVID and non-COVID related deaths per week
Gorges and Konetzka^22^	13312	50	1 January 2020-september 13, 2020	CMS	Association between the racial composition of nursing home residents and COVID-19 deaths	Case fatality rate from COVID-19, measured as number of deaths per 100 COVID-19 cases
Kim et al.^36^	117	IL (single county)	1 January 2020- Sept 30, 2020	State reports	Relationships among relevant resident, facility, and community characteristics to estimate the total effects of independent variables on COVID-19 deaths	Cumulative COVID-19 deaths
Wang^32^	686	FL	July 26, 2020, and October 25, 2020	CMS	Relationship between quality ratings and infection control citations with COVID-19 deaths. Changes in COVID-19 deaths in FL over time.	Binary variable: was there a COVID-19 resident death in the facility?
Weech-Maldonado et al.^14^	12,914	50	Through 25 October 2020	CMS	Relationship between nursing home racial/ethnic mix and COVID-19 mortality	Binary variable: was there a COVID-19 resident death in the facility?
Khairat et al.^20^	14,690	50	May 25, 2020-December 20, 2020	CMS	Relationship between CMS Five Star Quality Rating and COVID-19 deaths	Rate of excess deaths, calculated as (total confirmed cases or deaths)-[(confirmed case or death rate at 5-star NHs) * total resident-weeks
Kumar et al.^19^	11,718	50	June 1, 2020- Dec 23, 2020	CMS	Relationship between NH resident race and mortality rate, longitudinally	Mortality rate: COVID-19 deaths per 1000 residents
Simoni-Wastila et al.^16^	13,154	50	Sept 23, 2020- December 27, 2020	CMS	Relationship between potentially modifiable facility-level infection control factors on odds and magnitude of COVID-19 deaths in NH	Mortality rate: COVID-19 deaths per occupied beds
Williams et al.^13^	14,693	50	Through January 2021	CMS	Relationship between NH quality ratings and COVID-19 outbreak severity and persistence	Cumulative mortality rate: cumulative deaths per 1000 residents
Das Gupta et al.^26^	12,415	50	June 1, 2020- January 31, 2021	CMS	Relationship between NH quality and COVID-19 deaths over time	nursing home resident COVID-19 death rate (per 100,000 residents)
Yang^39^	14,693	50	May 25, 2020-February 28, 2020	CMS	Relationship between rural/urban status and COVID-19 deaths over time	Binary variable: was there a COVID-19 resident death in the facility?
Dean^25^	13,350	50	June 8, 2020- March 21, 2021	CMS	Relationship between union status and nursing home resident deaths	Cumulative count of deaths
Gilman and Bassett^23^	13,820	50	May 21, 2020-April 18, 2021	CMS	Relationship between resident race and COVID-19 death trends	Weekly COVID-19 deaths per 1000 residents
Cronin and Evans^27^	14,905	50	Through April 25, 2021	CMS	Relationship between nursing home quality and non-COVID-19 deaths	Cumulative COVID and non-COVID death rates per 100 beds
Iyanda and Boakye^21^	13,335	48	Through December 18, 2021	CMS	Relationship among nursing home characteristics and location with COVID-19 deaths	Cumulative COVID-19 death counts; cumulative COVID-19 deaths/ 100 bed days
Frochen et al.^40^	14,833	50	Through July 31, 2022	CMS	To test whether State Veteran’s Homes were associated with different infection and mortality rates than non-veteran nursing homes	Cumulative COVID-19 deaths
Lakon and Hipp	12,403	50	June 2020 through September 2022	CMS	Examine whether resident deaths relate to socio-spatial health disparities present in the areas surrounding the SNF	Cumulative COVID-19 deaths count
Wong et al.^35^	11,690	48	Through January 2022	CMS	What geospatial and racial characteristics are correlated with COVID-19 cases and deaths in nursing homes, and where are clusters of COVID-19 risk?	Cumulative COVID-19 death count

NH, nursing home; SVI, Social Vulnerability Index.

### Outcomes

Our study’s outcomes included whether the NH had at least one death, the number of deaths, death rates, and case fatality rates.

Eleven papers reported deaths as a rate compared with bed days or census. Six papers reported deaths as a binary measure of whether the NH reported at least one death. Eight papers reported a count of deaths, either during a specific period or as a cumulative number. Two studies included non-COVID-19 deaths in their outcomes.^17,27^ One study measured the case fatality rate or the number of COVID-19 deaths divided by the number of COVID-19 cases.^22^ See Table 1 for study outcomes.

### Place-based factors

Nine studies included place-based factors as the primary independent variables and all other studies included place-based factors as covariates. When place-based factors were covariates, statistics were rarely reported (Table 2). Table 3 shows the geographic variables identified in the included studies.

**Table 2.** Findings

	Place-based factors				
Study	Individual	Interpersonal/ organizational	Community	Societal	Findings
Temkin-Greener et al.^33^			Community viral spread		Positive association with chance of deaths. Likelihood of at least one death was increased in counties with highest level of COVID-19 (OR = 4.44, *p* < 0.001)
Dean et al.^30^			Community viral spread		Not statistically significant
LeRose et al.^8^			SVI		Positively associated with mortality. SNFs in the highest quartile for SVI had 1.86 times the mortality rate as compared to the lowest SVI quartile
Li et al.^37^	Income: median				Covariate, no statistics reported
	Age: % population over 65				Covariate, no statistics reported
	Education: % of population with high school or higher level of education				Covariate, no statistics reported
			Community spread		Covariate, no statistics reported
			Market competition		Association with mortality not reported. Positive association with NH with higher shares of minority residents
Li et al.^31^			Community spread		Covariate, no statistics reported
			County population		Covariate, no statistics reported
Gorges and Konetzka^38^			Community spread		Not statistically significant
			Urbanity: Rural Urban Continuum (RUCA)		No association with deaths
Travers et al.^15^			Community spread		Covariate, no statistics reported
			Urbanity: binary		No association between urbanity and deaths reported. Urbanity mediated the relationship between proportion of Black residents and COVID-19 deaths. Rural NH had a stronger association
Shen^34^	Income: % of county residents under poverty line				Not statistically significant
	Race: % of non-white county population				Not statistically significant
		Staff community measures: population density, public transportation use, and racial makeup of staff community			Staff public transportation use, staff neighborhood % non-white residents, and staff neighborhood COVID-19 viral spread were positively associated with COVID-19 deaths in the nursing home. Community characteristics of NH staff were more predictive than NH community characteristics
			Community spread		Positively associated with NH deaths (0.55, *p* > 0.001)
Cai et al.^28^	Income: county median				Very small negative association (−0.0002, *p* < 0.0001) with likelihood of death
	Age: % of population in zip code 65 or older				Not statistically significant
	Education: % of population with at least high school education				Not statistically significant
	Race: binary measure of high/low minority population				Very small (0.0002, *p* < 0.0001) positive association likelihood of death in high minority communities
			Community spread		Positively associated with probability of any COVID-19 death (0.019, *p* = .0.002)
			Urbanity: binary		Rural NH slightly negatively associated with COVID-19 deaths. The association was stronger in low-minority NH
Li et al.^17^			Community spread		Covariate, statistics not reported
			Population: Number of people residing in the county		Covariate, statistics not reported
				Policies: stringency of state distancing measures, on a 100-point scale	Higher stringency was associated with lower COVID-19 deaths but higher non-COVID-19 deaths. State policies had a stronger effect on NH with higher proportions of racial minority residents
Gorges and Konetzka^22^			Community spread		Moderated the association between racial mix of residents and number of deaths. Accounting for community spread reduced the strength of association between race and mortality
Kim et al.^36^			Community spread: zip code-level infection rate		No statistical significance
			SVI		No statistical significance
Wang^32^			Urbanity: binary		No statistical significance
Weech-Maldonado et al.^14^			Urbanity: binary		Increased risk of death associated with metro location
			SVI: Robert Graham Center		Increased risk of death with higher SVI
Khairat et al.^20^			Community spread		Covariate, statistics not reported
			MUA designation		Covariate, statistics not reported
	Income: county-level median household income				Covariate, statistics not reported
	Age: % population age >65 years				Covariate, statistics not reported
	Race: percentage non-Hispanic white population				Covariate, statistics not reported
	Education: % less than high school level				Covariate, statistics not reported
Kumar et al.^19^			Community prevalence		Covariate, statistics not reported
			Urbanity: RUCA		Covariate, statistics not reported
Simoni-Wastila et al.^16^			Community prevalence		Not statistically significant
Williams et al.^13^			Community prevalence		Positively associated with COVID-19 deaths
			Social vulnerability: measured area deprivation index		Not statistically significant
			Urbanity: binary		Covariate, statistics not reported
Das Gupta et al.^26^			Community prevalence		Most important covariate, statistics not reported
			Urbanity: binary		Covariate, statistics not reported
			Social Vulnerability: SVI		Covariate, statistics not reported
Yang^39^			Community prevalence		Covariate, statistics not reported
			Urbanity: binary		Higher risk of deaths associated with location in urban counties. Risk of deaths in urban NH was higher and stayed stable over time. Risk of deaths in rural NH increased over time, but remained lower than risk in urban
Dean^25^			Community prevalence		Covariate, statistics not reported
Gilman and Bassett^23^			Community prevalence		Covariate, statistics not reported; notes that community prevalence was a significant predictor
Cronin and Evans^27^			Community prevalence		Covariate, statistics not reported
				Politics: % Republican votes in 2016 election	Covariate, statistics not reported
Iyanda and Boakye^21^			Community prevalence		Covariate, statistics not reported
			Urbanity: RUCA		Urban/rural status moderated the relationship between NH quality and mortality
Frochen et al.^40^	County vaccination rate				Covariate, not statistically significant
			Community prevalence		Covariate, not statistically significant
			Pandemic Vulnerability Index (PVI)		Covariate, unexpected negative association with COVID-19 deaths when controlling for COVID-19 cases
Lakon and Hipp	Race: Percent minorities, racial heterogeneity in the ½ mile surrounding the NH				% racial minority was positively associated with deaths in 2020, negatively or not associated with deaths after Oct 2020. Racial heterogeneity was initially positively associated with more deaths, but not after Oct 2020
	Socioeconomic Status: average household income and income inequality in the ½ mile surrounding the NH				Income inequality was negatively associated with deaths after Oct 2020 but no association before oct 2020
Wong et al.^35^			Urbanity: RUCA		Rural nursing homes have lower deaths than metropolitan areas; micropolitan areas are not statistically different from metropolitan areas
				State	Deaths compared to Pennsylvania, which had the highest cumulative COVID-19 deaths. 38 states had statistically significant death rates than Pennsylvania

**Table 3.** Place-Based Factors

NIMHD level	Factor	Description	Findings
Individual*	Income	Measures of neighborhood resident income: median income, % living under poverty line, income inequality	Small, negative association between income and mortality. Small, inconsistent positive association between income inequality and mortality
	Age	Percent of the population over 65 years of age	No significant findings reported.
	Education	Percent of the population with high school or higher level of education	No significant findings reported.
	Race	Community racial makeup reported as % of minoritized community residents, high/low minoritized population, racial heterogeneity	Findings inconsistent across studies. Relationship between community racial makeup and NH mortality changed over time,^29^ or was found to be insignificant,^34^ or was associated with slightly higher risk of COVID-19 deaths in NH.^28^
	Vaccination	Percent of county residents with COVID-19 vaccination	No significant findings reported.
Organizational	Staff neighborhoods	Measures of population density, public transportation use, and racial makeup of the nursing home staff’s estimated neighborhoods	Staff neighborhood characteristics were more impactful than NH resident characteristics on NH mortality. Staff public transportation use, staff neighborhood % non-white residents, and staff neighborhood COVID-19 viral spread were positively associated with COVID-19 deaths in the nursing home.
Community	Community viral spread	New cases or rate of spread of COVID-19 in the neighborhood where a NH was located	Positive association with NH deaths.
	Social Vulnerability Indices	Composite measures of socioeconomic disadvantage of the community	Inconsistent results. Two studies found a positive association with NH deaths, one found no association, and one found a negative association.
	Urbanity	Either a binary measure of urban/metro location, or USDA rural-urban continuum codes	Metropolitan areas associated with higher risk of NH deaths. Urbanity moderated the relationship between race and COVID-19 mortality.
	Population	Number of county residents	No significant findings reported.
	Market forces	Measure of the level of competition for NH care	Mortality findings not reported.
	Medically underserved areas	A Health Resources and Services Administration measure of availability of medical providers for the local population	No significant findings reported.
Societal	Government policies	Strength of state distancing measures on a 100 point scale	Higher stringency associated with lower COVID-19 deaths but higher non-COVID-19 deaths.
	Political leaning	Percent of republican votes in 2016 election	No findings reported.
	State	U.S. State the NH is located in	Variations in death rates were found by state.

### Individual factors

Individual measures of place-based factors included income, age, education, race, and vaccination rate. Results were inconsistent across studies for income and race. Community age, education, and vaccination were not associated with mortality. Individual measures were aggregated as zip codes or county averages. Six studies included measures of individual-level community characteristics. Of these, three studies only included individual measures as control variables with no reported statistics.^20,37,40^

Cai et al. found a small increased risk of death in high minority communities and a small, negative association between income and mortality.^28^ Three studies found minimal or no statistically significant association between measured individual characteristics and NH mortality.^28,34,40^ Shen found that race and income were not associated with mortality. One study found that the relationships between individual local characteristics varied over time.^29^ There was a peak of increased deaths in communities with more significant numbers of racial minorities during the initial pandemic outbreak, but this trend was not consistent as of October 2020. Income inequality was positively associated with NH deaths before October 2020 but negatively related to deaths after October 2020.^29^

### Interpersonal/organizational

One study included an organizational place-based variable. In that study, the researchers attempted to estimate demographic information about the NH’s employees, including racial makeup, use of public transportation, and population density of their neighborhoods—these were positively associated with NH deaths.^34^ The community characteristics of NH staff were more strongly associated with NH mortality rates than the community characteristics of the NH itself.

### Community factors

Community factors identified in the sample studies included the community prevalence of COVID-19, social vulnerability indices, market competition, population density, urbanity, and medically underserved area status. No significant findings were reported for medically underserved area status or market competition.

Community prevalence of COVID-19 is any measure of the disease burden in the community surrounding an NH. Community prevalence was positively associated with NH deaths. Nineteen studies included a measure of community prevalence. Community prevalence was measured as county-level new case rates, county-level confirmed COVID-19 cases, or county-level COVID-19 death rates. In all studies, community prevalence was a control variable. Dean et al.^30^ found that community prevalence was not a statistically significant predictor of COVID-19 deaths. This may be due to low variation and overall high rates of COVID-19 in a study that included only New York. Another study found that community spread was not significant in their model. Gorges et al. found that community prevalence moderated the association between racial makeup of the NH residents and mortality.^22^ Shen found that the community prevalence in the NH staff’s neighborhood was four times more impactful on resident deaths than the community spread in the NH’s neighborhood.^34^ Simoni-Wastila found that community prevalence predicted NH cases, but not mortality.^16^ Two studies found that community prevalence was the most impactful control variable in their model.^13,26^

Overall, 12 studies included measures of urbanity. Eight studies treated urban/metro as a binary covariate. The U.S. Department of Agriculture rural-urban continuum codes were used in four studies. Five studies found that metropolitan areas were at higher risk of higher mortality rates. Other studies concluded that urbanity moderated COVID-19 outcomes and other disparity conditions. One study reported that the strength of the relationship between Black residents and COVID-19 deaths was stronger in rural NH.^15^ Another study found that NH with lower shares of minority residents tended to be in rural areas.^28^ Wong et al. found that while rural communities had lower deaths than metropolitan areas, communities in between likely have different experiences.^35^

Social vulnerability factors include scores that measure disparity factors in the local community. Examples include the Social Vulnerability Index (SVI) or area deprivation index.^41^ SVI includes measures of socioeconomic factors that inversely affect communities during emergencies, such as poverty, crowded housing, and lack of transportation availability.^42^ Higher scores indicate greater vulnerability or less favorable conditions.

Five studies included a composite measure, SVI,^8,26,36^ or another social deprivation index.^13,14^ Lerose et al. found a positive association between SVI and mortality; higher social vulnerability was associated with higher mortality. Weech-Maldonado et al. also found a positive correlation between SVI and mortality. Williams et al. found that SVI was associated with COVID-19 cases, but not deaths. One study included the Pandemic Vulnerability Index and found that pandemic vulnerability was negatively associated with NH deaths, but the result was not statistically significant.^40^

### Societal factors

We found three studies that included societal measures with conflicting results. One study compared each state and found significant differences in COVID mortality between the 48 contiguous states, with Pennsylvania having the most deaths.^35^ One study included the share of Republican votes in the 2016 election as a covariate but did not report statistics. The third study examined the stringency of state distancing measures, measured on a 100-point scale. Higher stringency was associated with fewer COVID-19 deaths, but a higher rate of non-COVID-19 deaths.^17^

## Discussion

This review aimed to identify place-based factors associated with mortality rates among NHs during the recent COVID-19 pandemic. We identified several consistent place-based factors—community spread and rurality—as well as multiple factors with inconsistent findings. Consistent with a previous literature review,^6^ community spread of the COVID-19 virus was the most consistent predictor of higher mortality rates in NHs and was included in most studies on mortality in NHs during the pandemic. However, we also identified other place-based factors that contributed to disparities among NH.

Rurality is the next most common place-based factor. While rurality is often associated with worse health status^43^ and worse care in NHs,^44^ most studies found that rurality was associated with lower mortality rates during the pandemic. We hypothesize that this is related to community spread and lower population density, which are important factors in a communicable disease pandemic.^45^ One study found that the socioeconomic characteristics of the staff’s neighborhood mattered more than the NH neighborhood, suggesting that supporting staff may be an important factor in protecting NH residents during disasters.^34^

We found conflicting results regarding community COVID-19 spread among the included studies. Some variation may be due to the chosen metrics (i.e., probability of at least one death vs. continuous measure of death rate). Longitudinal studies found changes in associations over time, suggesting that the most important factors associated with pandemic outcomes differed at the onset of the pandemic, as compared with months or years after the pandemic impacts. As the pandemic wore on, we may have seen changes due to successful policy implementation or that better-resourced NHs may have been “worn down” over time. This may explain the reduction in racial, economic, and rural disparities over time.

The United States has a long history of structural racism.^5^ Communities with increased numbers of minoritized residents often experience reduced health care access and economic and occupational inequalities.^46^ Some of these inequities are related to the long history of segregated communities.^47^ Outcomes based on racial disparity were mixed for the studies included in the review. Residence in an urban setting was a mediating factor in the relationship between race and COVID-19 mortality.^15,28^ Other studies found that racial makeup of staff and the local community affected NH mortality.^29,34^ In all cases, higher percentages of minoritized individuals, especially within Black communities, had a higher risk of death. These findings highlight the importance of data to inform policy and resourcing interventions intended to improve health equity in historically marginalized communities.

### Limitations

Due to the pace at which COVID-19 research was published, it is possible that the research team missed potentially relevant studies that were not found in the three databases included in our methods. The studies included in our study were associational studies that could not make causal claims. Limiting our search to COVID-19 pandemic studies may limit generalizability to other types of disasters or “normal” operations. However, our findings are useful for preparing for future pandemics.

### Future studies

Only one study included presence in a medically underserved area as a covariate. Medically underserved areas are locations without an adequate number of medical providers for the resident population. A small proportion of our studies included place-based factors in their research questions, even though place-based factors are an important component of structural racism and health disparities.^9,10^ Future research on NH disparities should address this gap, particularly as more sophisticated methods of addressing geographical contributors to health outcomes are available.^4^

Only two studies included non-COVID deaths as an outcome. Cai had an unexpected finding that non-COVID deaths were higher in high-quality NHs. More NH residents died from non-COVID causes in states with stronger social distancing policies. Therefore, more research is needed to understand the impact of place-based factors on overall mortality rates. Loneliness or other adverse outcomes due to social distancing requirements may have impacted non-COVID outcomes and should be investigated further.

### Policy implications

We found that location was an important factor in NH mortality; however, the lack of reported statistics in these studies makes it challenging to identify actionable policy implications. For this reason, researchers should be encouraged to report place-based disparity data through funding or prioritization mechanisms.

State-level variation in mortality suggests that health policy might significantly impact NH resident survival. Both social distancing policies and other health care-related policies, as well as political preferences, are related to COVID deaths both in the NH and in the general population.^45^

## Conclusions

All disasters are local.^48^ While the COVID-19 pandemic impacted the entire world, it impacted different locations with different severity. Place-based health disparities are an important consideration when designing policies and interventions to protect NH residents.

## Abbreviations Used

CMS: Centers for Medicare and Medicaid Services
MUA: Medically underserved areas
NIMHD: National Institute on Minority Health and Health Disparities
PRISMA: Preferred Repoing Items for Systematic reviews and Meta-Analyses
